# Paracrine actions of glucagon-like peptide 1 in the gut unraveled

**DOI:** 10.1093/lifemeta/loac010

**Published:** 2022-07-01

**Authors:** Camille Allard, Daniela Cota

**Affiliations:** University of Bordeaux, INSERM, Neurocentre Magendie, U1215 Bordeaux, France; University of Bordeaux, INSERM, Neurocentre Magendie, U1215 Bordeaux, France

**In the last issue of *Cell*, Zhang *et al.* comprehensively pinpointed in male mice how endogenous glucagon-like peptide 1 (GLP-1) production by intestinal L cells triggers a coordinated interorgan crosstalk to mediate stomach distension and appetite suppression.**

GLP-1 was originally described at the beginning of the 20th century as a peptide deriving from pre-proglucagon, and then officially named in the 1980s [1]. GLP-1 has a large number of metabolic actions, the most studied ones being its capacity to inhibit gastric emptying, suppress food intake, and reduce glycemia [1]. GLP-1 receptor (GLP-1R) agonists are effective drugs for the treatment of type 2 diabetes and obesity [1]. Consequently, there is extensive interest in understanding the precise mechanisms of actions of GLP-1. While initial studies had put forward a prevalent endocrine effect, in part because of the widespread expression of GLP-1R in the organism, subsequent work on GLP-1, by pointing out its very short half-life and very low levels in the blood, has challenged this view [2]. Rather, local action of GLP-1, primarily within the gut, may represent an important conduit of its relevance in physiology [3, 4].

**Figure 1:**
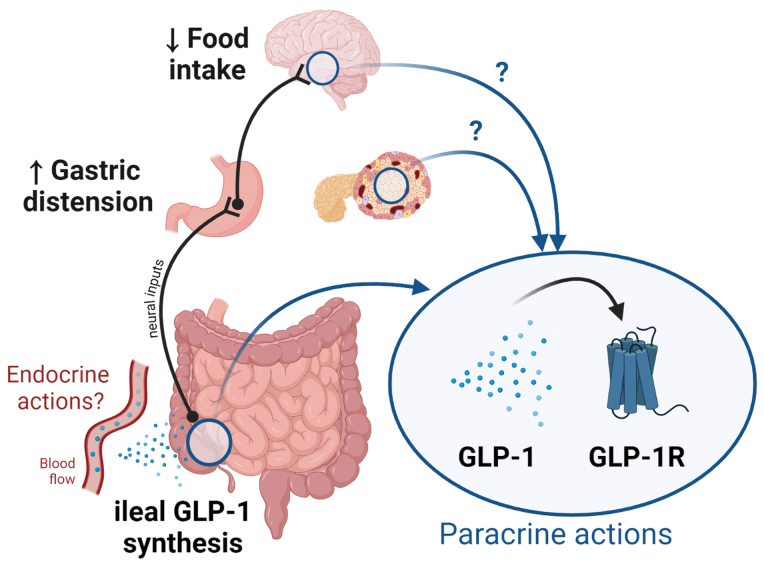
Local GLP-1 production by L cells recruits an interorgan neural circuit to mediate gastric and anorectic effects of GLP-1, demonstrating its paracrine, rather than endocrine action. The paracrine action of GLP-1 at the level of the brain and the pancreatic islets, two tissues containing pre-proglucagon synthetizing cells and GLP-1R, should be further investigated. The figure was created with BioRender.com.

Recently, Zhang *et al.* [5] provided further critical evidence of a paracrine action of GLP-1 by detailing how local GLP-1 production by L cells recruits an interorgan neural circuit to mediate gastric and anorectic effects of GLP-1 (Fig. 1). For this purpose, the authors went on to map GLP-1-dependent neuronal responses, from the local GLP-1 production site heading to different organs (stomach, via the celiac ganglion, toward the hypothalamus via the spinal cord, to finally reach the mouth musculature), by using an extensive amount of cutting-edge methods. They first described that ileal GLP-1 signaling induces gastric distension and appetite loss by combining optogenetics and chemogenetics strategies. To note, only the ileal injection of GLP-1 was able to relax stomach muscle, and thus induce distension, unlike the intraperitoneal or intracerebroventricular route. This effect was lost following local ablation of ileal GLP-1R^+^ cells. Next, they used retrograde-tracing approaches to demonstrate that activation of intestinofugal enteric GLP-1R^+^ neurons recruited stomach-innervating sympathetic neurons via celiac ganglion activation, as the GLP-1-induced effects vanished after celiactomies. They further showed that gastric neurons expressing the neuronal nitric oxide (Nos1) are crucial for the bloating and anorectic effects of ileal GLP-1. Then, they investigated how ileal GLP-1-dependent signals are transmitted to the brain. It has been mostly admitted that vagal sensory afferents are at play during GLP-1 anorectic action [6]. In the present study, the authors focused on the sensory tract and showed that gastric distension signals induced by GLP-1 convey to the brain via spinal, rather than vagal, sensory neurons. This observation suggests that ileal GLP-1 signaling drives coordinated autonomic neural responses for regulating feeding behavior and gastric emptying [7]. Thanks to *in vivo* behavioral tests, the authors finally showed that the reduction of food intake induced by ileal GLP-1 was mediated through activation of the parasubthalamic nucleus of the lateral hypothalamus and projections to craniofacial musculatures to induce food rejection.

Altogether, these findings allow precisely pinpointing the physiological roles of ileal GLP-1, which the authors show to be critical for the ileal brake reflex, a mechanism triggered by nutrient malabsorption, rather than for the physiological satiety response. Indeed, although ileal GLP-1 infusions decreased food intake, ileal GLP-1R antagonism did not increase food intake. In addition, they demonstrated that the anorectic action of ileal GLP-1 is due to stomach distension, since the reduction in food intake requires enteric gastric neurons. Finally, this work remarkably shows that sensory spinal neurons, rather than vagal afferents convey the ileal GLP-1-dependent nutrient malabsorption signal to the brain. Thus, spinal sensory neurons importantly contribute to gastric interoception. Of note, especially when considering that a common side effect of GLP-1R agonists in the clinic is nausea, this study also shows that intestinal GLP-1 actions were independent of the expression of GLP-1R in the area postrema of the brainstem, a structure implicated in GLP-1-induced nausea. Thus, targeting specific GLP-1R^+^ cell populations would bypass the nausea responses commonly associated with GLP-1R agonists. The formidable work carried out by Zhang and colleagues also paves the way to a number of questions. First, is there a role for intestinal GLP-1 in physiological satiety? Based on the evidence provided, this does not seem the case for ileal GLP-1. However, a possibility, also mentioned by the authors, is that proximal gut GLP-1 release may be relevant for satiety. This possibility will require further study. Second, is there a role for ileal GLP-1 in glucose metabolism? Of note, Zhang and colleagues did not observe any change in blood glucose after ileal GLP-1 administration. However, GLP-1 insulinotropic effects may be explained by its gut anti-motility properties [8]. Accordingly, the recent demonstration that proglucagon-derived gut peptides, including GLP-1, decrease glucose levels [3], may be explained by taking into account their anti-motility action. This consideration also brings back onto the table the paracrine nature of GLP-1 action, since previous studies had shown that local pancreatic GLP-1 expression is necessary to mediate GLP-1 insulinotropic actions in pancreatic islets [9]. Third, and related to the above, is there any physiological role for circulating GLP-1? Based on current [5] and past evidence [3, 9], one could wonder whether variation of circulating plasma GLP-1 concentration could be a “spillover” of locally produced GLP-1 rather than a “true” endocrine signal (Fig. 1). This is why local brain production of GLP-1 should be further reconsidered and investigated [10].
